# A comparison of trunk circumference and width indices for hypertension and type 2 diabetes in a large-scale screening: a retrospective cross-sectional study

**DOI:** 10.1038/s41598-018-31624-x

**Published:** 2018-09-05

**Authors:** Bum Ju Lee, Boncho Ku

**Affiliations:** Korea Institute of Oriental Medicine, Future Medicine Division, Deajeon, 305-811 Republic of Korea

## Abstract

Anthropometric indices determine important risk factors for many chronic diseases. However, to date, no study has simultaneously analyzed the capabilities of trunk circumference and width indices to identify hypertension and type 2 diabetes in a large-scale screening study. The objectives of this study were to examine the associations of hypertension and - diabetes with circumference and width indices measured at the five identical positions (axillary, chest, rib, waist, and pelvic) and to compare the capabilities of circumference and width indices to identify the two diseases. Data were obtained from the Korean Health and Genome Epidemiology Study database. The associations and abilities of the circumference indices to identify diabetes were greater than those for hypertension. Overall, trunk circumference indices displayed stronger associations with and greater abilities to identify hypertension and diabetes than did trunk width indices at the five positions. In the comparative analysis between index pairs of circumference and width in patients with diabetes, significant differences were shown at all five positions and in the adjusted analysis of axillary, chest, rib, and pelvic positions. Therefore, width indices should not be used as an alternative indicator of type 2 diabetes in either men or women, except when measured at the waist.

## Introduction

The prevalence of overweight and obesity is increasing, and obesity is a common risk factor for all-cause mortality worldwide^1–4^. Anthropometric indices related to obesity have been shown to indicate important risk factors for hypertension, type 2 diabetes, cardiovascular diseases, metabolic syndrome, coronary heart disease, certain forms of cancer, and sleep-breathing disorders^1,2,5^. Several previous studies have discussed the best predictors of individual or general chronic diseases among various obesity-related anthropometric indices across diverse ethnic groups, genders, and countries^1,2,6^. For example, subjects with large thigh or hip circumferences have a low risk of type 2 diabetes, and individuals with a large abdominal circumference have a high risk of type 2 diabetes, regardless of age, gender, waist circumference (WC), and body mass index (BMI)^7^. In addition, increased WC, waist-to-hip ratio (WHR), BMI, and waist-to-thigh ratio are strongly correlated with type 2 diabetes and hypertension^8,9^. Therefore, anthropometric indices related to obesity are considered very important indicators of risk factors for hypertension and type 2 diabetes.

An effort was undertaken to determine the international standard of anthropometric indices by comparison of anthropometric indices with fat-free mass and visceral adipose tissue under the consideration of posture, respiration, and fasting state^10–14^. For the use of WC worldwide, the position of measurement, posture, respiration, and fasting state should be standardized to develop an international standard protocol for the measurement of WC^10^. The association of WC, BMI, and waist-to height ratio (WHtR) with percentage body fat (measured by dual-energy X-ray absorptiometry) in a large, nationally representative US population based on several race-ethnicity groups was examined^11^, and the study suggested that WC, WHtR, and BMI perform similarly as alternative measurements of body fatness. Additionally, the correlations between visceral adipose tissue measured by whole-body MRI and anthropometric indices such as waist circumference and waist-hip ratio were determined to prove the usefulness of DXA-based abdominal region of interest (ROI) indices^14^.

Despite the large number of published studies on the associations between anthropometric indices and many chronic diseases, studies on the associations of simultaneously measured anthropometric circumference and width indices with chronic diseases have very rarely been reported. In other words, trunk circumference and ratio indices based on circumference have mainly been used to predict hypertension^15–19^, diabetes^7,9,20–25^, cancers^26^, chronic kidney disease^27^, cardiovascular diseases^28–33^, and other diseases^34–36^, whereas trunk width indices have rarely been used to identify these diseases^9^. Furthermore, to date, no study has simultaneously analyzed the capabilities of trunk circumference and width indices to predict and identify hypertension and type 2 diabetes in Korean adults. The primary objectives of the present study were to examine the associations of hypertension and type 2 diabetes with circumference and width indices and to compare the capabilities of circumference and width indices to identify the two diseases. To the best of our knowledge, this study is the first to simultaneously examine the abilities of circumference and width indices to predict hypertension and type 2 diabetes in Korea. Our findings may provide clinical information for initial screens for hypertension and type 2 diabetes in a large-scale screening study.

## Material and Methods

### Study population and data source

The data were obtained from the Korean Health and Genome Epidemiology Study (KHGES) database and the Korea Institute of Oriental Medicine (KIOM). All participants were recruited from multi-center hospitals in 27 rural and urban areas, consisting of Ansan, Anseong, and other areas in the Republic of Korea, from November 2016 to August 2007. The present study was performed according to the standards of the International Committee on Harmonization on Good Clinical Practice and the revised version of the Declaration of Helsinki. All subjects participated voluntarily and provided written informed consent for participation in this study. The KIOM Institutional Review Board (IRB) approved this study (No. I-1210/002/002-02), and this study was performed according to the relevant guidelines and regulations by the IRB of the KIOM as well as the Korea University Ansan Hospital (AS10153), the Ajou University Hospital (AJIRB-MED-SUR-12-377), and all TKM hospitals.

**Figure 1:**
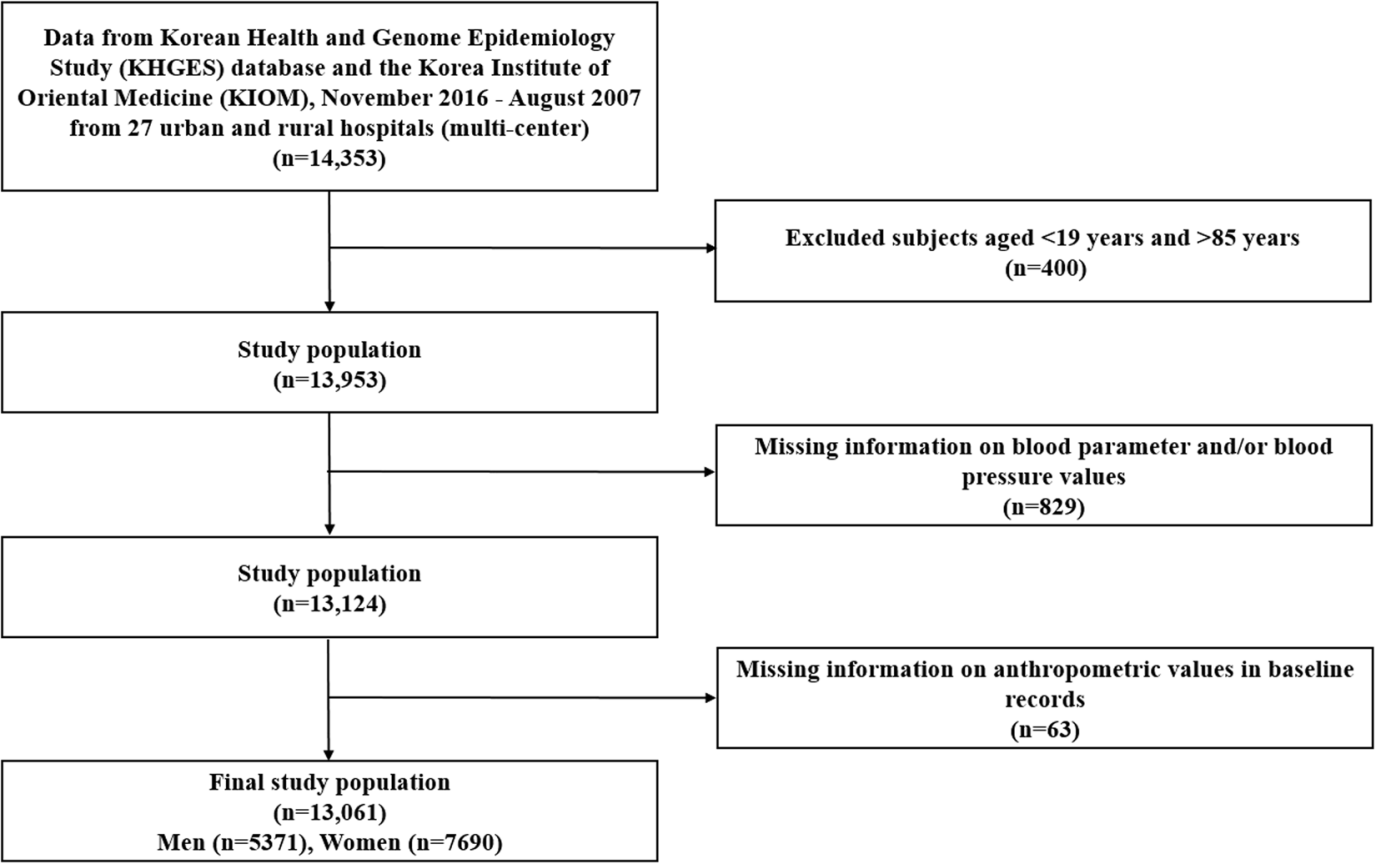
Sample selection procedure.

The following exclusion criteria were applied for sample selection: participants with (1) missing values for blood parameters, trunk anthropometric indices, and/or blood pressure; (2) missing information for basic characteristics, such as gender, age, education, and/or region; and (3) missing values for other important data. The inclusion criteria were: participants who (1) provided written informed consent; (2) were aged 19–85 years; and (3) were Koreans residing in the Republic of Korea. Finally, 13,061 participants (5,371 men and 7,690 women aged 19–85 years) were included in this retrospective cross-sectional study. Figure 1 presents a detailed description of the sample selection procedure.

### Definition

We used the criteria from the 1990 World Health Organization (WHO) report^37^ and the American Association of Clinical Endocrinologists (AACE)^38^ to diagnose type 2 diabetes and the criteria from the 1999 WHO^39^ and the 2013 European Society of Hypertension-European Society of Cardiology guidelines for the management of arterial hypertension (grade 1 hypertension) to diagnose hypertension^40^. Therefore, hypertension was defined as physician-diagnosed hypertension, systolic blood pressure (SBP) ≥140 mmHg, and/or diastolic blood pressure (DBP) ≥90 mmHg. Type 2 diabetes was defined as physician-diagnosed type 2 diabetes and/or a fasting plasma glucose (FPG) level of ≥110 mg/dl. If subjects had been diagnosed with type 2 diabetes or hypertension by a physician in the past but had completely recovered, they were considered normal subjects. Among a total of 14,353 subjects who participated in this retrospective cross-sectional study, 25 subjects in the hypertensive patients group were fully cured (men 12, women 13), and 5 patients with type 2 diabetes were fully cured (men 2, women 3). In this study, “completely recovered” means that the doctors diagnosed the patients as no longer needing medication or treatment at the time of data collection.

### Measurement

Blood samples were obtained after a minimum 8-hour fast for the measurement of FPG levels using standardized protocols (ADVIA1800, Siemens, USA). Demographic data, such as gender, age, education, and region of residence, were documented by administering a questionnaire to all participants. Well-trained observers or physicians measured all anthropometric indices, including weight, height, and trunk circumferences and widths, to the nearest 0.1 kg or 0.1 cm, respectively, of all participants wearing lightweight clothing without shoes using standardized protocols (LG-150; G Tech International Co., Ltd., Uijeongbu, Republic of Korea). Circumferences and widths of the axillary, chest, rib, waist, and pelvic positions were measured using 6 tapelines (150 cm/60 inches, Hoechstmass, Germany) and six large sliding calipers (50 cm/20 inches, Samhwa, Korea)^41^. For example, waist circumference was measured at the level of the umbilicus using a non-elastic tape, and waist width was measured as the horizontal distance between the left and right sides at the level of the umbilicus at the front of the subjects using sliding calipers. Detailed descriptions of the five measurement positions and methods have been provided in previous studies^25,41,42^. The basic characteristics and a brief description of all indices used in this study for patients of each gender are described in Table 1. Comparisons of the characteristics between both men and women in the normal group and the two disease groups are presented in Table 2.

**Table 1:** Basic characteristics of variables and trunk positions used in this study.

Variable	Men	Women	P-value
N (%)	5371 (41.1%)	7690 (58.9%)	
Age (year)	53.7 ± 13.7	52.8 ± 14.0	<0.001
Height (cm)	168.3 ± 6.3	155.9 ± 6.2	<0.0001
Weight (kg)	68.9 ± 10.3	57.8 ± 8.4	<0.0001
Systolic BP (mmHg)	122.2 ± 15.5	118.5 ± 16.7	<0.0001
Diastolic BP (mmHg)	80.2 ± 10.7	76.6 ± 10.7	<0.0001
Pulse (bpm)	68.8 ± 9.6	69.7 ± 9.4	<0.0001
Circumference indices (cm)			
AxillaryC	95.7 ± 6.2	87.8 ± 6.2	<0.0001
ChestC	93.9 ± 6.6	90.4 ± 8.0	<0.0001
RibC	87.8 ± 6.8	79.6 ± 8.3	<0.0001
WaistC	87.2 ± 8.2	83.9 ± 9.3	<0.0001
PelvicC	91.4 ± 6.4	90.4 ± 7.3	<0.0001
HipC	93.6 ± 5.9	92.9 ± 6.0	<0.0001
Ratio indices			
WHR	0.93 ± 0.06	0.90 ± 0.07	<0.0001
WHtR	0.52 ± 0.05	0.54 ± 0.07	<0.0001
BMI (kg/m^2^)	24.3 ± 3.0	23.8 ± 3.4	<0.0001
Width indices (cm)			
AxillaryW	32.9 ± 2.4	30.8 ± 2.4	<0.0001
ChestW	30.1 ± 2.5	29.7 ± 3.1	<0.0001
RibW	29.4 ± 2.4	27.3 ± 2.4	<0.0001
WaistW	28.8 ± 2.9	28.0 ± 3.1	<0.0001
PelvicW	29.0 ± 2.3	29.2 ± 2.5	<0.0001
Laboratory test			
AST (U/L)	27.8 ± 16.9	23.7 ± 10.6	<0.0001
ALT (U/L)	27.9 ± 18.4	21.3 ± 14.3	<0.0001
BUN (mg/dL)	15.7 ± 4.5	14.4 ± 4.3	<0.0001
Creatinine (mg/dL)	1.1 ± 0.2	0.8 ± 0.2	<0.0001
Fasting plasma glucose (mg/dL)	102.0 ± 25.6	96.6 ± 24.2	<0.0001
Triglycerides (mg/dL)	150.9 ± 110.1	120.9 ± 72.9	<0.0001
Total cholesterol (mg/dL)	186.4 ± 34.6	192.0 ± 34.8	<0.0001
HDL cholesterol (mg/dL)	44.6 ± 11.7	50.7 ± 13.4	<0.0001
LDL cholesterol (mg/dL)	113.5 ± 32.5	116.0 ± 32.3	<0.0001
Hemoglobin (g/dL)	14.9 ± 1.2	12.9 ± 1.1	<0.0001
Hematocrit (%)	44.2 ± 3.6	38.2 ± 3.2	<0.0001
Education			<0.0001
Uneducated	159 (3.0%)	764 (9.9%)	
Elementary school	683 (12.7%)	1561 (20.3%)	
Middle school	836 (15.6%)	1122 (14.6%)	
High school	1956 (36.4%)	2411 (31.4%)	
University	1389 (25.9%)	1539 (20.0%)	
More than graduated school	348 (6.5%)	293 (3.8%)	

Continuous variables are summarized as the mean ± SD and categorical variables as the frequency (%). P-values are derived from independent two-sample tests for continuous variables and χ^2^ tests for categorical variables. Abbreviations: BP, blood pressure; WHR, waist-to-hip ratio; WHtR, waist-to-height ratio; BMI, body mass index; AST, aspartate transaminase; ALT, alanine transaminase; L/HDL, low/high density lipoprotein; C., circumference; W., width.

Axillary W., chest W., rib W., waist W., and pelvic W. were measured by horizontal distances between the right axilla and left axilla, between the right side and left side at the chest position, between the right side and left side at the rib position, between the right side and left side at the waist position, and between the right side and left side at the pelvic position, respectively, using calipers.

**Table 2:** Characteristics of men and women stratified into normal and hypertension or type 2 diabetes groups.

Variable	Men			Women			Men			Women		
	Normal	Hypertension	P-value	Normal	Hypertension	P-value	Normal	Diabetes	P-value	Normal	Diabetes	P-value
N (%)	3,759 (70.0%)	1,612 (30.0%)	—	5,940 (77.2%)	1,750 (22.8%)	—	4,265 (79.4%)	1,106 (20.6%)	—	6,672 (86.8%)	1,018 (13.2%)	—
Age (year)	53.3 ± 14.0	54.7 ± 13.0	<0.001	51.0 ± 14.1	59.0 ± 11.7	<0.0001	52.5 ± 14.2	58.5 ± 10.1	<0.0001	51.5 ± 13.9	61.2 ± 11.3	<0.0001
Height (cm)	168.4 ± 6.3	168.2 ± 6.4	0.295	156.3 ± 6.1	154.5 ± 6.1	<0.0001	168.5 ± 6.5	167.7 ± 5.7	<0.001	156.2 ± 6.2	153.9 ± 5.8	<0.0001
Weight (kg)	68.0 ± 10.0	70.9 ± 10.9	<0.0001	57.3 ± 8.1	59.5 ± 9.1	<0.0001	68.4 ± 10.4	70.6 ± 10.0	<0.0001	57.5 ± 8.2	59.9 ± 9.3	<0.0001
Systolic BP (mmHg)	115.9 ± 11.1	136.8 ± 14.6	<0.0001	112.8 ± 12.2	137.7 ± 15.9	<0.0001	121.3 ± 15.3	125.7 ± 15.8	<0.0001	117.5 ± 16.5	125.2 ± 16.5	<0.0001
Diastolic BP (mmHg)	75.9 ± 7.7	90.2 ± 9.9	<0.0001	73.2 ± 8.2	88.0 ± 10.2	<0.0001	79.9 ± 10.8	81.2 ± 10.2	<0.001	76.3 ± 10.6	78.8 ± 10.8	<0.0001
Pulse (bpm)	68.1 ± 9.2	70.4 ± 10.3	<0.0001	69.3 ± 9.2	71.2 ± 10.1	<0.0001	68.2 ± 9.4	70.8 ± 10.0	<0.0001	69.3 ± 9.3	72.6 ± 9.7	<0.0001
AxillaryC (cm)	95.0 ± 6.0	97.4 ± 6.5	<0.0001	87.1 ± 6.0	90.1 ± 6.3	<0.0001	95.3 ± 6.2	97.3 ± 6.1	<0.0001	87.3 ± 6.1	90.9 ± 6.1	<0.0001
ChestC (cm)	93.1 ± 6.3	95.8 ± 6.8	<0.0001	89.5 ± 7.7	93.7 ± 8.1	<0.0001	93.4 ± 6.5	96.1 ± 6.3	<0.0001	89.8 ± 7.8	94.9 ± 7.7	<0.0001
RibC (cm)	86.9 ± 6.6	90.0 ± 6.8	<0.0001	78.4 ± 7.9	83.5 ± 8.1	<0.0001	87.1 ± 6.8	90.5 ± 6.3	<0.0001	78.8 ± 8.0	84.8 ± 7.9	<0.0001
WaistC (cm)	86.2 ± 8.0	89.6 ± 8.2	<0.0001	82.8 ± 9.0	87.9 ± 9.3	<0.0001	86.4 ± 8.1	90.3 ± 7.9	<0.0001	83.2 ± 9.1	89.0 ± 9.0	<0.0001
PelvicC (cm)	90.7 ± 6.2	92.8 ± 6.7	<0.0001	89.7 ± 7.1	93.1 ± 7.4	<0.0001	90.9 ± 6.4	93.0 ± 6.2	<0.0001	90.0 ± 7.2	93.5 ± 7.3	<0.0001
HipC (cm)	93.0 ± 5.6	94.9 ± 6.3	<0.0001	92.5 ± 5.8	94.4 ± 6.5	<0.0001	93.4 ± 5.9	94.2 ± 5.8	<0.0001	92.8 ± 6.0	94.0 ± 6.4	<0.0001
WHR (ratio)	0.93 ± 0.06	0.94 ± 0.05	<0.0001	0.89 ± 0.07	0.93 ± 0.07	<0.0001	0.92 ± 0.06	0.96 ± 0.5	<0.0001	0.90 ± 0.07	0.95 ± 0.07	<0.0001
WHtR (ratio)	0.51 ± 0.05	0.53 ± 0.05	<0.0001	0.53 ± 0.06	0.57 ± 0.06	<0.0001	0.51 ± 0.05	0.54 ± 0.05	<0.0001	0.53 ± 0.06	0.58 ± 0.06	<0.0001
BMI (kg/m^2^)	23.9 ± 2.9	25.0 ± 3.1	<0.0001	23.5 ± 3.3	24.9 ± 3.4	<0.0001	24.1 ± 3.0	25.0 ± 3.0	<0.0001	23.6 ± 3.3	25.3 ± 3.4	<0.0001
AxillaryW (cm)	32.8 ± 2.4	33.1 ± 2.5	<0.0001	30.7 ± 2.4	31.3 ± 2.4	<0.0001	32.9 ± 2.4	33.0 ± 2.5	0.160	30.7 ± 2.4	31.5 ± 2.5	<0.0001
ChestW (cm)	29.8 ± 2.4	30.7 ± 2.6	<0.0001	29.4 ± 3.0	30.7 ± 3.1	<0.0001	30.0 ± 2.5	30.5 ± 2.5	<0.0001	29.5 ± 3.0	31.0 ± 3.1	<0.0001
RibW (cm)	29.1 ± 2.3	30.1 ± 2.5	<0.0001	27.1 ± 2.4	28.2 ± 2.5	<0.0001	29.3 ± 2.4	29.9 ± 2.3	<0.0001	27.2 ± 2.4	28.3 ± 2.5	<0.0001
WaistW (cm)	28.5 ± 2.8	29.7 ± 2.9	<0.0001	27.6 ± 3.0	29.0 ± 3.1	<0.0001	28.6 ± 2.9	29.6 ± 2.9	<0.0001	27.8 ± 3.0	29.1 ± 3.1	<0.0001
PelvicW (cm)	28.8 ± 2.3	29.4 ± 2.4	<0.0001	29.1 ± 2.5	29.7 ± 2.6	<0.0001	29.0 ± 2.4	29.2 ± 2.2	<0.001	29.2 ± 2.5	29.5 ± 2.4	<0.0001
AST (U/L)	27.1 ± 15.9	29.2 ± 18.9	<0.001	23.3 ± 10.1	25.0 ± 11.9	<0.0001	27.3 ± 16.5	29.3 ± 18.3	<0.05	23.4 ± 10.2	25.6 ± 12.6	<0.0001
ALT (U/L)	27.0 ± 16.9	30.1 ± 21.3	<0.0001	20.7 ± 13.2	23.2 ± 17.2	<0.0001	27.3 ± 18.8	30.2 ± 16.5	<0.0001	20.7 ± 13.9	25.3 ± 15.9	<0.0001
BUN (mg/dL)	15.6 ± 4.3	15.8 ± 4.7	0.132	14.2 ± 4.1	15.0 ± 4.5	<0.0001	15.5 ± 4.3	16.4 ± 5.1	<0.0001	14.2 ± 4.1	15.5 ± 4.9	<0.0001
Creatinine (mg/dL)	1.0 ± 0.2	1.1 ± 0.2	<0.001	0.8 ± 0.2	0.9 ± 0.2	<0.0001	1.0 ± 0.2	1.1 ± 0.3	<0.05	0.8 ± 0.2	0.9 ± 0.2	<0.0001
Fasting plasma glucose (mg/dL)	100.9 ± 24.3	104.5 ± 28.3	<0.0001	94.7 ± 20.0	103.0 ± 33.9	<0.0001	93.0 ± 8.6	136.6 ± 37.4	<0.0001	90.4 ± 8.3	137.2 ± 45.3	<0.0001
Triglycerides (mg/dL)	144.5 ± 105.7	165.8 ± 118.5	<0.0001	114.4 ± 68.6	143.1 ± 82.0	<0.0001	142.7 ± 94.7	182.8 ± 151.9	<0.0001	115.5 ± 67.6	156.2 ± 93.7	<0.0001
Total Cholesterol (mg/dL)	185.3 ± 33.7	189.0 ± 36.3	<0.001	190.9 ± 34.4	195.6 ± 36.1	<0.0001	186.9 ± 34.1	184.5 ± 36.4	<0.05	191.8 ± 34.6	193.4 ± 35.9	0.181
HDL cholesterol (mg/dL)	44.7 ± 11.8	44.3 ± 11.6	0.189	51.6 ± 13.4	47.8 ± 12.8	<0.0001	45.4 ± 11.8	41.7 ± 10.8	<0.0001	51.4 ± 13.4	46.0 ± 12.3	<0.0001
LDL cholesterol (mg/dL)	112.7 ± 31.0	115.3 ± 35.8	<0.05	115.2 ± 31.9	118.7 ± 33.4	<0.001	114.2 ± 31.6	110.8 ± 35.9	<0.05	115.8 ± 31.9	117.3 ± 34.6	0.192
Hemoglobin (g/dL)	14.9 ± 1.2	15.0 ± 1.3	<0.05	12.9 ± 1.1	13.0 ± 1.2	<0.0001	15.0 ± 1.2	14.7 ± 1.4	<0.0001	12.9 ± 1.1	13.0 ± 1.2	<0.05
Hematocrit (%)	44.2 ± 3.6	44.3 ± 3.7	0.359	38.1 ± 3.2	38.4 ± 3.4	<0.001	44.4 ± 3.5	43.7 ± 4.1	<0.0001	38.1 ± 3.2	38.4 ± 3.5	<0.05

Mean ± SD. These results indicate significant difference between the normal and hypertension groups and the normal and type 2 diabetes groups of men and women, as separately analyzed using independent Student’s t-tests.

Abbreviations: BP, blood pressure; WHR, waist-to-hip ratio; WHtR, waist-to-height ratio; BMI, body mass index; AST, aspartate transaminase; ALT, alanine transaminase; L/HDL, low/high density lipoprotein; C., circumference; W., width.

### Statistical analysis

All statistical analyses used to examine associations and predictive power were performed with SPSS 23 for Windows (SPSS Inc., Chicago, IL, USA). A binary logistic regression analysis was conducted to determine the significance of differences between the normal group and the hypertension group and between the normal group and the type 2 diabetes group after transforming all data in a standardized manner in both the crude analysis and the analysis adjusted for age, area of residence, and education. From two independent logistic models corresponding to the pair of circumference and width indices, the magnitude of the difference between beta coefficients related to circumference and width measure was statistically tested using the simple Z test^43^ under the assumption that those two models were independent. We considered the area under the receiver operating characteristic (ROC) curve as the criterion for the comparison of the predictive abilities of trunk circumference and width indices to identify the two diseases because ROC curves are widely used to examine the predictive power of indices and diagnostic accuracy in medicine and biological research.

## Results

### Associations of hypertension with trunk circumference and width indices

Of the 5,371 men analyzed, the numbers (proportions) of patients with hypertension and type 2 diabetes were 1,612 (30%) and 1,106 (20.6%), respectively. Of the 7,690 women, the numbers (proportions) with hypertension and type 2 diabetes were 1,750 (22.8%) and 1,018 (13.2%), respectively. In general, the anthropometric indices measured at the five identical positions (i.e., axillary, chest, rib, waist, and pelvic) displayed a stronger association with the two diseases and a better predictive ability in women than in men. The associations and abilities of circumference indices to predict type 2 diabetes were higher than those for hypertension in both men and women. Additionally, trunk circumference indices tended to have a higher association with hypertension and type 2 diabetes than did trunk width indices in crude and adjusted analysis in both men and women. Tables 3 and 4 present associations of hypertension with circumference indices and width indices in men and women, respectively.

**Table 3:** Associations of hypertension with circumference and width indices in men.

	Crude				Adjusted			
	Ratio $\exp \,({\hat{{\boldsymbol{\beta }}}}_{{\boldsymbol{R}}})$	C. $\exp \,({\hat{{\boldsymbol{\beta }}}}_{{\boldsymbol{C}}})$	W. $\exp \,({\hat{{\boldsymbol{\beta }}}}_{{\boldsymbol{W}}})$	C. − W.^†^ ${\hat{{\boldsymbol{\beta }}}}_{{\boldsymbol{C}}}-{\hat{{\boldsymbol{\beta }}}}_{{\boldsymbol{W}}}$	Ratio $\exp \,({\hat{{\boldsymbol{\beta }}}}_{{\boldsymbol{R}}})$	C. $\exp \,({\hat{{\boldsymbol{\beta }}}}_{{\boldsymbol{C}}})$	W. $\exp \,({\hat{{\boldsymbol{\beta }}}}_{{\boldsymbol{W}}})$	C. − W.^†^ ${\hat{{\boldsymbol{\beta }}}}_{{\boldsymbol{C}}}-{\hat{{\boldsymbol{\beta }}}}_{{\boldsymbol{W}}}$
Axillary	—	1.48*** (1.39, 1.57)	1.14*** (1.07, 1.20)	0.262***	—	1.52*** (1.42, 1.62)	1.39*** (1.29, 1.49)	0.088
Chest	—	1.51*** (1.42, 1.60)	1.43*** (1.34, 1.51)	0.056	—	1.52*** (1.43, 1.63)	1.46*** (1.37, 1.56)	0.042
Rib	—	1.62*** (1.52, 1.72)	1.50*** (1.41, 1.60)	0.074	—	1.58*** (1.48, 1.69)	1.47*** (1.37, 1.57)	0.072
Waist	—	1.54*** (1.45, 1.64)	1.54*** (1.45, 1.64)	0.004	—	1.53*** (1.43, 1.63)	1.50*** (1.41, 1.61)	0.019
Pelvic	—	1.40*** (1.32, 1.49)	1.28*** (1.20, 1.35)	0.093*	—	1.44*** (1.35, 1.53)	1.20*** (1.12, 1.29)	0.178**
WHR	1.37*** (1.29, 1.46)	—	—	—	1.47*** (1.37, 1.58)	—	—	—
WHtR	1.56*** (1.47, 1.66)	—	—	—	1.58*** (1.48, 1.69)	—	—	—
BMI	1.44*** (1.35, 1.53)	—	—	—	1.57*** (1.47, 1.68)	—	—	—

Crude odds ratio ($\exp ({\hat{\beta }}_{\ast })$) and adjusted odds ratio ($\exp ({\tilde{\beta }}_{\ast })$) for residence, age, and education level and their 95% confidence intervals, where *={C., W.}.

*p < 0.05, **p < 0.001, ***p < 0.0001.

^†^Wald test for difference between two beta coefficients from independent models.

Abbreviations: C., circumference; W., width.

**Table 4:** Associations of hypertension with circumference and width indices in women.

	Crude				Adjusted			
	Ratio $\exp \,({\hat{{\boldsymbol{\beta }}}}_{{\boldsymbol{R}}})$	C. $\exp \,({\hat{{\boldsymbol{\beta }}}}_{{\boldsymbol{C}}})$	W. $\exp \,({\hat{{\boldsymbol{\beta }}}}_{{\boldsymbol{W}}})$	C. − W. ${\hat{{\boldsymbol{\beta }}}}_{{\boldsymbol{C}}}-{\hat{{\boldsymbol{\beta }}}}_{{\boldsymbol{W}}}$	Ratio $\exp \,({\hat{{\boldsymbol{\beta }}}}_{{\boldsymbol{R}}})$	C. $\exp \,({\hat{{\boldsymbol{\beta }}}}_{{\boldsymbol{C}}})$	W. $\exp \,({\hat{{\boldsymbol{\beta }}}}_{{\boldsymbol{W}}})$	C. − W. ${\hat{{\boldsymbol{\beta }}}}_{{\boldsymbol{C}}}-{\hat{{\boldsymbol{\beta }}}}_{{\boldsymbol{W}}}$
Axillary	—	1.63*** (1.55, 1.73)	1.29*** (1.22, 1.36)	0.235***	—	1.51*** (1.42, 1.61)	1.44*** (1.36, 1.54)	0.047
Chest	—	1.70*** (1.61, 1.80)	1.56*** (1.48, 1.65)	0.084*	—	1.53*** (1.43, 1.63)	1.44*** (1.35, 1.53)	0.062
Rib	—	1.85*** (1.75, 1.95)	1.55*** (1.47, 1.64)	0.176***	—	1.51*** (1.42, 1.61)	1.42*** (1.34, 1.51)	0.063
Waist	—	1.76*** (1.67, 1.86)	1.59*** (1.51, 1.68)	0.102*	—	1.49*** (1.40, 1.59)	1.43*** (1.34, 1.52)	0.044
Pelvic	—	1.60*** (1.51, 1.69)	1.28*** (1.22, 1.35)	0.222***	—	1.42*** (1.34, 1.51)	1.26*** (1.18, 1.35)	0.119*
WHR	1.69*** (1.60, 1.79)	—	—	—	1.39*** (1.29, 1.49)	—	—	—
WHtR	1.84*** (1.74, 1.94)	—	—	—	1.54*** (1.43, 1.65)	—	—	—
BMI	1.52*** (1.44, 1.60)	—	—	—	1.51*** (1.42, 1.61)	—	—	—

Crude odds ratio ($\exp ({\hat{\beta }}_{\ast })$) and adjusted odds ratio ($\exp ({\tilde{\beta }}_{\ast })$) for residence, age, and education level and their 95% confidence intervals, where *={C., W.}.

*p < 0.05, **p < 0.001, ***p < 0.0001.

^†^Wald test for difference between two beta coefficients from independent models.

Abbreviations: C., circumference; W., width.

In men, RibC had the strongest association with hypertension among all circumference and width indices (odds ratio (OR) = 1.62 [95% CI, 1.52, 1.72], adjusted OR = 1.58 [1.48, 1.69], and ROC = 0.63 [0.61, 0.65]). However, the magnitude of the association was similar to that of the associations of hypertension with WHtR and BMI among ratio indices in adjusted analysis. Among the width indices, WaistW showed the highest association with hypertension (OR = 1.54 [1.45, 1.64], adjusted OR = 1.5 [1.41, 1.61], and ROC = 0.62 [0.6, 0.64]), but its predictive power was lower than that of RibC. WaistC, which is one of the most widely used indices (OR = 1.54 [1.45, 1.64], adjusted OR = 1.53 [1.43, 1.63], and ROC = 0.62 [0.6, 0.64]), and WaistW showed similar abilities to predict hypertension in men. In comparative analysis between index pairs of circumference and width at the five identical positions, significant differences were shown in axillary and pelvic positions in the crude analysis (p < 0.0001 and p < 0.05, respectively). In adjusted analysis, a significant difference was shown in pelvic position (p < 0.001).

In women, hypertension was the most strongly associated with RibC (OR = 1.85 [1.75, 1.95], adjusted OR = 1.51 [1.42, 1.61], and ROC = 0.68 [0.66, 0.69]) and WHtR (OR = 1.84 [1.74, 1.94], adjusted OR = 1.54 [1.43, 1.65], and ROC = 0.67 [0.66, 0.69]) among all circumference and width indices in the crude analysis. But after the adjustment of confounders, these associations were similar to those of AxillaryC, ChestC, and BMI. When comparing the indices measured at the five identical positions (i.e., axillary, chest, rib, waist, and pelvic), the predictive power of the width indices was generally lower than that of the circumference indices in women. In comparative analysis between index pairs of five identical positions, significant differences between circumference and width were shown at all five positions in the crude analysis. However, in the adjusted analysis, a significant difference was observed only at the pelvic position (p < 0.05).

### Associations of type 2 diabetes with trunk circumference and width indices

Tables 5 and 6 present associations of type 2 diabetes with anthropometric circumference indices and width indices in men and women, respectively. Also, Table 7 presents the comparison of the power of all individual indices to identify hypertension and type 2 diabetes.

**Table 5:** Associations of type 2 diabetes with circumference and width indices in men.

	Crude				Adjusted			
	Ratio $\exp \,({\hat{{\boldsymbol{\beta }}}}_{{\boldsymbol{R}}})$	C. $\exp \,({\hat{{\boldsymbol{\beta }}}}_{{\boldsymbol{C}}})$	W. $\exp \,({\hat{{\boldsymbol{\beta }}}}_{{\boldsymbol{W}}})$	C. − W. ${\hat{{\boldsymbol{\beta }}}}_{{\boldsymbol{C}}}-{\hat{{\boldsymbol{\beta }}}}_{{\boldsymbol{W}}}$	Ratio $\exp \,({\hat{{\boldsymbol{\beta }}}}_{{\boldsymbol{R}}})$	C. $\exp \,({\hat{{\boldsymbol{\beta }}}}_{{\boldsymbol{C}}})$	W. $\exp \,({\hat{{\boldsymbol{\beta }}}}_{{\boldsymbol{W}}})$	C. − W. ${\hat{{\boldsymbol{\beta }}}}_{{\boldsymbol{C}}}-{\hat{{\boldsymbol{\beta }}}}_{{\boldsymbol{W}}}$
Axillary	—	1.39*** (1.30, 1.48)	1.05 (0.98, 1.12)	0.278***	—	1.61*** (1.49, 1.73)	1.38*** (1.27, 1.49)	0.153*
Chest	—	1.51*** (1.41, 1.62)	1.21*** (1.14, 1.29)	0.219***	—	1.63*** (1.52, 1.76)	1.45*** (1.34, 1.56)	0.121*
Rib	—	1.66*** (1.55, 1.79)	1.32*** (1.24, 1.42)	0.229***	—	1.67*** (1.55, 1.80)	1.51*** (1.40, 1.62)	0.106
Waist	—	1.63*** (1.52, 1.74)	1.43*** (1.34, 1.53)	0.129*	—	1.62*** (1.50, 1.74)	1.60*** (1.49, 1.72)	0.010
Pelvic	—	1.37*** (1.29, 1.47)	1.12** (1.05, 1.19)	0.207***	—	1.43*** (1.33, 1.54)	1.19*** (1.11, 1.28)	0.183**
WHR	1.88*** (1.74, 2.02)	—	—	—	1.72*** (1.59, 1.86)	—	—	—
WHtR	1.71*** (1.59, 1.83)	—	—	—	1.58*** (1.47, 1.70)	—	—	—
BMI	1.38*** (1.29, 1.47)	—	—	—	1.55*** (1.45, 1.67)	—	—	—

Crude odds ratio ($\exp ({\hat{\beta }}_{\ast })$) and adjusted odds ratio ($\exp ({\tilde{\beta }}_{\ast })$) for residence, age, and education level and their 95% confidence intervals, where *={C., W.}.

*p < 0.05, **p < 0.001, ***p < 0.0001.

^†^Wald test for difference between two beta coefficients from independent models.

Abbreviations: C., circumference; W., width.

**Table 6:** Associations of type 2 diabetes with circumference and width indices in women.

	Crude				Adjusted			
	Ratio $\exp \,({\hat{{\boldsymbol{\beta }}}}_{{\boldsymbol{R}}})$	C. $\exp \,({\hat{{\boldsymbol{\beta }}}}_{{\boldsymbol{C}}})$	W. $\exp \,({\hat{{\boldsymbol{\beta }}}}_{{\boldsymbol{W}}})$	C − W ${\hat{{\boldsymbol{\beta }}}}_{{\boldsymbol{C}}}-{\hat{{\boldsymbol{\beta }}}}_{{\boldsymbol{W}}}$	Ratio $\exp \,({\hat{{\boldsymbol{\beta }}}}_{{\boldsymbol{R}}})$	C. $\exp \,({\hat{{\boldsymbol{\beta }}}}_{{\boldsymbol{C}}})$	W. $\exp \,({\hat{{\boldsymbol{\beta }}}}_{{\boldsymbol{W}}})$	C. − W. ${\hat{{\boldsymbol{\beta }}}}_{{\boldsymbol{C}}}-{\hat{{\boldsymbol{\beta }}}}_{{\boldsymbol{W}}}$
Axillary	—	1.76*** (1.65, 1.89)	1.39*** (1.30, 1.49)	0.238***	—	1.61*** (1.50, 1.73)	1.42*** (1.32, 1.52)	0.128*
Chest	—	1.89*** (1.76, 2.02)	1.63*** (1.53, 1.74)	0.147*	—	1.65*** (1.53, 1.78)	1.51*** (1.40, 1.62)	0.088
Rib	—	2.03*** (1.90, 2.17)	1.52*** (1.42, 1.62)	0.292***	—	1.68*** (1.56, 1.82)	1.46*** (1.36, 1.57)	0.141*
Waist	—	1.87*** (1.74, 2.00)	1.54*** (1.44, 1.65)	0.191***	—	1.51*** (1.40, 1.63)	1.44*** (1.34, 1.55)	0.050
Pelvic	—	1.60*** (1.50, 1.71)	1.14*** (1.07, 1.22)	0.341***	—	1.37*** (1.27, 1.47)	1.17*** (1.08, 1.26)	0.160*
WHR	2.10*** (1.95, 2.25)	—	—	—	1.64*** (1.51, 1.79)	—	—	—
WHtR	1.98*** (1.85, 2.12)	—	—	—	1.54*** (1.42, 1.67)	—	—	—
BMI	1.60*** (1.50, 1.71)	—	—	—	1.48*** (1.38, 1.59)	—	—	—

Crude odds ratio ($\exp ({\hat{\beta }}_{\ast })$) and adjusted odds ratio ($\exp ({\tilde{\beta }}_{\ast })$) for residence, age, and education level and their 95% confidence intervals, where *={C., W.}.

*p < 0.05, **p < 0.001, ***p < 0.0001.

^†^Wald test for difference between two beta coefficients from independent models.

Abbreviations: C., circumference; W., width.

**Table 7:** Comparison of the ROC values of all individual indices in predicting hypertension and type 2 diabetes in men and women.

Index	Hypertension		Diabetes	
	Men	Women	Men	Women
AxillaryC	0.61 (0.59, 0.63)	0.64 (0.63, 0.66)	0.60 (0.58, 0.62)	0.67 (0.65, 0.68)
ChestC	0.61 (0.59, 0.63)	0.65 (0.64, 0.67)	0.63 (0.61, 0.64)	0.69 (0.67, 0.70)
RibC	0.63 (0.61, 0.65)	0.68 (0.66, 0.69)	0.65 (0.63, 0.67)	0.71 (0.69, 0.73)
WaistC	0.62 (0.60, 0.64)	0.66 (0.65, 0.68)	0.64 (0.62, 0.66)	0.68 (0.66, 0.70)
PelvicC	0.59 (0.57, 0.61)	0.63 (0.62, 0.65)	0.60 (0.58, 0.61)	0.64 (0.62, 0.66)
AxillaryW	0.54 (0.52, 0.56)	0.58 (0.56, 0.59)	0.52 (0.50, 0.54)	0.60 (0.58, 0.61)
ChestW	0.60 (0.58, 0.62)	0.64 (0.62, 0.65)	0.56 (0.54, 0.58)	0.65 (0.63, 0.67)
RibW	0.61 (0.60, 0.63)	0.63 (0.62, 0.65)	0.58 (0.56, 0.60)	0.63 (0.61, 0.64)
WaistW	0.62 (0.60, 0.64)	0.63 (0.62, 0.65)	0.60 (0.58, 0.62)	0.63 (0.61, 0.64)
PelvicW	0.57 (0.55, 0.59)	0.57 (0.56, 0.59)	0.54 (0.52, 0.56)	0.55 (0.53, 0.56)
WHR	0.59 (0.57, 0.61)	0.65 (0.63, 0.66)	0.67 (0.65, 0.68)	0.71 (0.69, 0.72)
WHtR	0.62 (0.61, 0.64)	0.67 (0.66, 0.69)	0.65 (0.63, 0.67)	0.70 (0.69, 0.72)
BMI	0.60 (0.59, 0.62)	0.63 (0.61, 0.64)	0.60 (0.58, 0.62)	0.64 (0.62, 0.66)

Abbreviations: C., circumference; W., width; ROC, receiver operating characteristic curve; WHR, waist-to-hip ratio; WHtR, waist-to-height ratio; BMI, body mass index.

In men, RibC was the most strongly associated with type 2 diabetes among circumference and width indices RibC (OR = 1.66 [1.55, 1.79], adjusted OR = 1.67 [1.55, 1.8], and ROC = 0.65 [0.63, 0.67]). However, in the comparison of circumference, width, and ratio indices, WHR had the strongest association with type 2 diabetes (OR = 1.88 [1.74, 2.02], adjusted OR = 1.72 [1.59, 1.86], and ROC = 0.67 [0.65, 0.68]). When comparing the indices measured at the five identical positions on the body, the width indices showed lower predictive power than did several circumference indices in the crude analysis. In the crude comparative analysis between index pairs of circumference and width, significant differences were shown for all five positions. In the adjusted analysis, significant differences were shown at the axillary, chest, and pelvic positions (p < 0.05, p < 0.05, and p < 0.001, respectively).

In women, type 2 diabetes had the strongest association with RibC (OR = 2.03 [1.9, 2.17], adjusted OR = 1.68 [1.56, 1.82], and ROC = 0.71 [0.69, 0.73]) and WHR (OR = 2.1 [1.95, 2.25], adjusted OR = 1.64 [1.51, 1.79], and ROC = 0.71 [0.69, 0.72]). Consistent with the results obtained for men, circumference indices measured at the five positions exhibited higher predictive power than did the width indices. In the comparative analysis between each pair, significant differences were shown for all five positions in the crude analysis. In the adjusted analysis, significant differences were shown at the axillary, rib, and pelvic positions (p < 0.05, p < 0.05, and p < 0.05, respectively). Therefore, width indices should not be used as an alternative predictor of type 2 diabetes in either men or women, except for at the waist position.

## Discussion

According to many epidemiological studies, strategies designed to prevent type 2 diabetes mellitus, hypertension, and cardiovascular diseases are important to control modifiable risk factors, such as obesity, visceral adiposity, physical activity, diet, and health-related quality of life^6,8,44–46^. For example, the Diabetes Prevention Program Research Group^46^ has documented that lifestyle changes implemented through a modification program producing a weight loss of approximately 7% and 150 minutes of physical activity per week decrease the incidence of diabetes in subjects at high risk. Furthermore, scientists argue that lifestyle changes are more helpful than the administration of 850 mg of metformin twice daily. Thus, anthropometric indices related to obesity are important for predicting hypertension and type 2 diabetes.

Previous studies on the associations of trunk circumference and width indices with hypertension and diabetes are very rare. Chuang and colleagues^9^ examined the associations of type 2 diabetes with trunk circumference and width indices among subjects with three different body sizes based on BMI. The magnitudes of the associations of type 2 diabetes with waist and breast circumferences were higher than those of the associations of the disease with waist and breast widths^9^. In a simple comparison of width and circumference indices, Pintér and colleagues^47^ suggested accurate estimation equations to calculate the visceral fat area, abdominal fat area, and subcutaneous fat area based on trunk width, circumference, and skinfold measurements in Hungarians. The magnitude of the correlations between hip circumference and visceral fat area, abdominal fat area, and subcutaneous fat area were higher than the correlations between hip width and the three fat areas^47^. Moreover, Wells and colleagues^48^ examined correlations between three-dimensional photonic scanning (3D-PS) and manual measurements of width and circumference of the chest and waist in several ethnic groups of children aged 5–11 years. Higher correlations were observed between 3D-PS and manual measurements of chest and waist circumferences than between 3D-PS and manual measurements of chest and waist widths in all ethnic groups^48^. Our findings are consistent with the results of a previous study^9^, indicating that trunk circumference indices display higher predictive power for type 2 diabetes than do trunk width indices.

Regarding the associations of anthropometric indices with hypertension and type 2 diabetes in various countries and ethnic groups, many studies have attempted to discover the best indicators of hypertension and diabetes among several anthropometric indices because cutoff values of obesity-related indices for the prediction of these diseases may differ, particularly in comparisons of Asians with white Caucasians^49–52^. For example, WC was the best indicator of hypertension in Canadian^28^, American^53^, Italian^19,54^, and Brazilian women^55^ in several studies. Additionally, the strongest predictors of hypertension were WHtR in Mauritian Creole men^16^, Hong Kong Chinese^31^, and meta-analyses^29,56^, and WHR was the strongest in Argentina (Caucasian migrants)^17^, adult Tehranian men^30^, and Australian adults^32^ in other studies. BMI was the strongest indicator of hypertension in Mauritian Indian women^16^, a lean Chinese population^18^, and a northern Chinese population^20^. WC was the strongest predictor of type 2 diabetes in a US population^53^, Brazilian women^55^, a northern Chinese population^20^, US males^21,57^, and German women^58^, whereas the strongest indicator of the disease was BMI in Pima Indians^23^. The best predictors of type 2 diabetes were WHtR in German men^58^ and meta-analyses^29,56^, and WHR was the strongest in adult Tehranian men^30^, Hong Kong Chinese^31^, and Australian adults^32^ in some studies. On the other hand, two or more indices among several obesity-related indices display equal predictive powers or should be considered simultaneously to predict type 2 diabetes or hypertension^22,24,33,59^. For example, Janssen and colleagues^59^ argued that the integrated use of WC and BMI in clinical practice was more useful than the use of one index because the WC cutoff predicts the diseases within several BMI categories. As shown in the meta-analysis by Vazquez and colleagues^22^, WC, BMI, and WHR predictors are similarly associated with diabetes. In a comparison of the results from the present study and those from previous studies, our finding that WHR was the best indicator of type 2 diabetes in men was consistent with the results of previous studies^30–32^ and our previous study^60^; however, our finding that RibC was the strongest predictor of hypertension was not consistent with the results of almost all previous studies. One of the reasons for this discrepancy was that few previous studies considered RibC in associations between anthropometric indices and hypertension. The finding that RibC was a strongest indicator of hypertension is consistent with our previous study^15^. Moreover, the finding that WHtR was the strongest predictor of hypertension was consistent with the results of previous studies^16,29,31,56^.

The present study has several limitations that must be considered. First, we could not establish cause-effect relationships due to the cross-sectional design. Our findings were limited by the retrospective cross-sectional study without longitudinal follow-up and intervention. Therefore, further study is needed to conduct more rigorous analyses and to determine predictive abilities by longitudinal follow-up and intervention. Second, our findings were limited to Korean adults because we enrolled only Korean subjects in this study, and many countries and ethnic populations utilize different cutoff values for BMI, WC, and body shapes.

In conclusion, in the present study, we examined the associations of hypertension and type 2 diabetes with trunk circumference and width indices measured at five trunk positions (i.e., axillary, chest, rib, waist, and pelvic). Moreover, the associations and abilities of trunk circumference indices to identify hypertension and type 2 diabetes were greater than those of trunk width indices in the Korean population. Therefore, width indices should not be used as alternative predictors of type 2 diabetes in either men or women, except for at the waist position. Our findings may provide clinical information for the initial screening of hypertension and type 2 diabetes in epidemiology and public health.

## Data Availability

Data are available from the Korean Health and Genome Epidemiology Study (KHGES) database Institutional Data Access/Ethics Committee and the Korea Institute of Oriental Medicine (KIOM) Korean medicine data center (KDC, http://kdc.kiom.re.kr/html/, permission number: 20130903–20140327) for researchers who meet the criteria for access to confidential data.
